# The Role of Caspases in Melanoma Pathogenesis

**DOI:** 10.3390/cimb46090562

**Published:** 2024-08-28

**Authors:** Agnieszka Szmurło, Klaudia Dopytalska, Michał Szczerba, Elżbieta Szymańska, Alicja Petniak, Marcin Kocki, Janusz Kocki, Irena Walecka

**Affiliations:** 1 Department of Dermatology, The National Institute of Medicine of the Ministry of the Interior and Administration, 02-507 Warsaw, Poland; 2 Department of Dermatology, Centre of Postgraduate Medical Education, 02-507 Warsaw, Poland; 3 Department of Clinical Genetics, Medical University of Lublin, 20-080 Lublin, Poland

**Keywords:** melanoma, caspase, caspase-3, pathogenesis

## Abstract

Melanoma (malignant melanoma, MM) is an aggressive malignant skin cancer with an increasing incidence rate. The complete pathogenesis of MM in not clear. Due to DNA damage, mutations, dysregulation of growth factors, inactivation of tumor suppressor genes, and activation of oncogenes, excessive uncontrolled growth of abnormal melanocytes occurs in melanomas. Caspases are a group of proteolytic enzymes that participate in several processes important in regulating mechanisms at the cellular level. They play a role in cell homeostasis and programmed cell death (apoptosis) and in the regulation of non-apoptotic cell death processes. Dysregulation of caspase activation plays a role in the etiology of cancers, including melanoma. Caspases can initiate and execute apoptosis and are involved in regulating cell death and controlling tumor growth. These enzymes also inhibit tumor growth by cleaving and inactivating proteins that are involved in cell proliferation and angiogenesis. Moreover, caspases are involved in the activation of immune processes through the processing and presentation of tumor antigens, which facilitates recognition of the tumor by the immune system. The role of caspases in melanoma is complex, and they may inhibit melanoma growth and progression. This work aims to review the current knowledge of the role of individual caspases in melanoma pathogenesis.

## 1. Introduction

Melanoma (malignant melanoma, MM) is the deadliest and most aggressive malignant skin cancer, with an ever-increasing incidence rate. Major risk factors include genetic alterations (e.g., BRAF and KIT mutations) and overexposure to ultraviolet (UV) radiation [1,2]. As a result of DNA damage, creation of mutations, dysregulation of growth factors, inactivation of tumor suppressor genes, and activation of oncogenes, excessive uncontrolled growth of abnormal melanocytes occurs. Several key molecular pathways are involved in this process, including the mitogen-activated protein kinase (MAPK) pathway, the RAF/MEK/ERK pathway, the phosphatidylinositol-3-kinase (PI3Ks) pathway, the protein kinase B (AKT) pathway, and the p53 pathway [2,3,4].

The mainstay of melanoma treatment is surgical excision and for inoperable stage III or IV MM, immunotherapy or molecularly targeted therapy [5,6]. Despite significant advances in research on the molecular aspects of MM pathogenesis and new therapeutic options, advanced-stage melanomas still have a high mortality rate, and the complete pathogenesis of melanoma requires further study.

Caspases are a group of proteolytic enzymes belonging to cysteine endoproteases. They participate in several processes important in regulating mechanisms at the cellular level through, among other things, their ability to inactivate proteins in the process of proteolysis [7,8,9]. In cells, these enzymes exist in an inactive form as so-called procaspases, built from a C-terminal small subunit, a large subunit, and an N-terminal prodomain. Proteolysis of the linker located between the small and large subunits is required for caspase activation. The N-terminal fragments of initiator caspases contain caspase recruitment domains (CARDs; caspase-1, caspase-2, caspase-4, caspase-5, caspase-9, and caspase-11) or death effector domains (DEDs; caspase-8 and caspase-10) that promote their recruitment and activation in multiprotein complexes [7,8,9]. Caspases play a key role in cell homeostasis and programmed cell death (apoptosis). In addition to apoptosis, these enzymes are involved in the regulation of necroptosis, pyroptosis, and autophagy, which are categorized as non-apoptotic cell death processes [8,10]. Dysregulation of caspase activation plays a role in the etiology of many diseases, such as cancer, autoimmune diseases, and neurodegenerative diseases [8,9].

The classification of caspases according to their role is widely recognized—enzymes involved in apoptosis processes include caspase-3, caspase-6, caspase-7, caspase-8, and caspase-9, while caspase-1, caspase-4, caspase-5, caspase-11, and caspase-12 are involved in the regulation of immune processes [8,10]. For many years, caspases were divided into “apoptotic” and “pro-inflammatory”. This classification remains useful to some extent, but according to recent knowledge, most “apoptotic” caspases have been assigned at least one non-apoptotic function. In contrast, “non-apoptotic” caspases, such as caspase-1, caspase-4, and caspase-5, induce “pyroptosis”, a form of death associated with massive activation of inflammatory cells. Within the group of “apoptotic” caspases, we can distinguish “initiators” and “apical” caspases versus “executors” and “effectors” or downstream” caspases. Caspases that initiate apoptosis are caspase-8, caspase-9, and caspase-10, in turn activating caspases that carry out apoptosis (caspase-3, caspase-6, and caspase-7). In turn, the “initiators” are divided into those involved in the extrinsic pathway of apoptosis (caspase-8 and caspase-10) and the intrinsic pathway (caspase-9) [11].

**Scheme 1:**
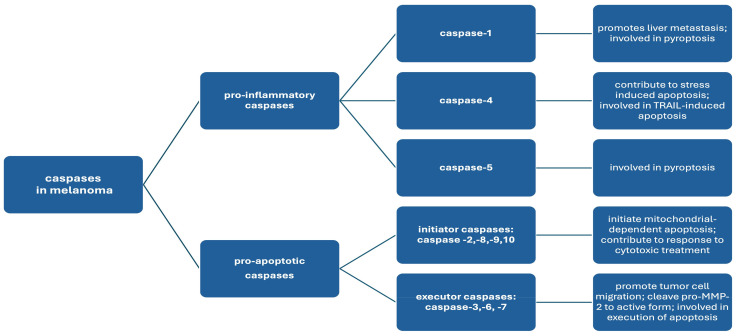
The classification of caspases according to their mechanism of action and role in melanoma pathogenesis.

In the context of malignant tumor pathogenesis, caspases are involved in regulating cell death and controlling tumor growth [12,13]. Among the key properties of caspases that may be important in the etiopathogenesis of malignant tumors is the ability to initiate and execute apoptosis. In cells where oncogenic mutations or DNA damage has occurred, caspases, through the stimulation of apoptosis, have an inhibitory effect on tumor growth [12,13]. These enzymes also inhibit tumor growth by cleaving and inactivating proteins that are involved in cell proliferation and angiogenesis. Importantly, caspases are also involved in the activation of immune processes through the processing and presentation of tumor antigens, which facilitates recognition of the tumor by the immune system. These enzymes also affect the production of pro-inflammatory cytokines [9,12,13]. Studies have shown that caspases also play significant roles in the effectiveness of anticancer treatment [13]. The role of caspases in melanoma is complex and dependent on many factors. Individual caspases are involved in different pathogenetic mechanisms of melanoma, depending on the MM stage, genetic alterations, and microenvironmental factors. This work aims to review the current knowledge of the role of individual caspases in melanoma pathogenesis [Scheme 1].

## 2. The Role of Specific Caspases in Melanoma Pathogenesis

### 2.1. Caspase-1, Caspase-4, and Caspase-5

Caspase-1, also known as interleukin-β-converting enzyme (ICE), is an initiator caspase that cleaves inactive pro-interleukin-1β to produce the active pro-inflammatory cytokine interleukin-1β [14]. Caspase-4, however, is a member of the inflammatory caspase subfamily and is implicated in endoplasmic reticulum (ER)-stress-induced apoptosis [15]. Caspase-1, caspase-4, and caspase-5 are crucial for immune system function and are also involved in inducing pyroptosis, a form of cell death associated with extensive activation of inflammatory cells [11,16].

Studies have shown that caspase-1 plays a significant role in melanoma metastasis. Vidal-Vanaclocha et al. demonstrated that both caspase-1 and interleukin-18 (IL-18) contribute to the development of melanoma liver metastasis. In their study, mice with mutations that prevented the production of IL-1β or caspase-1 exhibited fewer metastases compared to wild-type mice. IL-1β and IL-18 enhance the expression of adhesion molecules, like VCAM-1, facilitating melanoma cell attachment to blood vessel walls and promoting their migration to other organs [17]. Moreover, elevated levels of caspase-1 have been correlated with increased tumor mass and resistance to treatment in melanoma patients [14]. Mouawad et al. observed higher serum levels of caspase-1 in patients with metastatic malignant melanoma and found a positive correlation between caspase-1 levels, tumor mass, and resistance to re-treatment [14]. The precise mechanism behind the increased expression of caspase-1 in treatment-resistant patients remains unclear. Ramadani et al. hypothesized that this increase could be due to paracrine stimulation, as seen in chronic pancreatitis, or that cleaved caspase-1 is inactive and thus unable to induce apoptosis in cancer cells [18]. Further research is required to clarify these pathways.

Interleukin-1β (IL-1β) is known to promote inflammation, angiogenesis, and tissue remodeling and also plays a role in immune response regulation [7]. Okamoto et al. described advanced-stage melanoma cells exhibiting features of autoinflammatory diseases that spontaneously secreted active IL-1β. This occurs due to the activation of the NALP3 inflammasome and IL-1 receptor signaling. They showed that in melanoma cells, the NALP3 inflammasome is activated by caspase-1 cleavage, leading to continuous caspase activation in metastatic melanoma cells. Western blot analysis confirmed the presence of activated fragments of caspase-1 and caspase-5. Autoinflammation significantly contributes to melanoma progression, suggesting that inhibiting the inflammasome pathway or reducing IL-1 activity could be a potential therapeutic strategy for melanoma patients [19].

Caspase-1, caspase-4, caspase-5, and caspase-11 are also involved in pyroptosis, a pro-inflammatory form of regulated cell death. The executor of pyroptosis is gasdermin D (GSDMD)—a key protein in pyroptosis is cleaved by these caspases, primarily caspase-1 but also caspase-4, caspase-5, and caspase-11 [8,9,20,21]. Once cleaved, GSDMD forms pores in the cell membrane, leading to cell lysis and the release of pro-inflammatory cytokines like IL-1β. Pyroptosis is characterized by an intense inflammatory response due to the release of these mediators. Recent research indicates that gasdermin E (GSDME) can also be cleaved and activated by caspase-3, triggering a shift from apoptosis to pyroptosis [21].

Lou et al. developed a model to assess pyroptosis as a potential prognostic indicator in melanoma patients. Their study identified three pyroptosis-related genes—CASP1, CASP4, and PYCARD—that could predict the efficacy of anti-PD-1 immunotherapy in melanoma [21]. Pyroptosis has also been linked to tumor resistance to treatment and patient prognosis. It is proposed that targeting pyroptosis could enhance immunotherapy effectiveness, as pyroptosis is positively correlated with immune infiltration and immune checkpoint blockade biomarkers. Moreover, the levels of pyroptotic factors could serve as potential prognostic biomarkers in cancer immunotherapy [21]. Pyroptosis-related genes, including those encoding caspase-1, caspase-4, caspase-5, and caspase-11, are central to the pyroptosis process, activating GSDMD and leading to cell lysis and the release of pro-inflammatory cytokines like IL-1β. PEG genes, which influence pyroptosis, are being investigated as potential targets for anticancer therapies, as inducing pyroptosis in cancer cells, such as melanoma cells, may offer a novel therapeutic strategy to selectively destroy tumors. In conclusion, inducing pyroptosis in melanoma cells may represent a promising approach for selectively targeting the tumor [20,21].

Caspase-4 is closely linked to the endoplasmic reticulum (ER) stress response, particularly in melanoma cells. The induction of ER stress, such as by the TNF-related apoptosis-inducing ligand (TRAIL), leads to caspase-4 activation, which triggers apoptosis. Mao et al. demonstrated that caspase-4 activation in melanoma cells is associated with TRAIL-induced apoptosis. This process involves a cascade of caspase activation, where caspase-4 activation subsequently activates caspase-9 and caspase-3, both crucial for executing apoptosis [15]. Furthermore, the overexpression of Bcl-2 inhibits the activation of caspase-4, caspase-9, and caspase-3 induced by TRAIL. Caspase-3 inhibition blocks caspase-4 activation, and conversely, caspase-4 inhibition reduces TRAIL-stimulated caspase-3 activation, suggesting a reciprocal activation loop between these caspases. Mao et al. also found that TRAIL activates caspase-4 later in the apoptotic cascade, following the activation of caspase-8, caspase-9, and caspase-3. Importantly, TRAIL-induced ER stress, evidenced by specific stress markers, is necessary for caspase-4 activation, further solidifying the link between ER stress and apoptosis in melanoma [15]. The findings suggest that therapeutic strategies aimed at enhancing ER-stress-induced apoptotic pathways could potentiate the effectiveness of TRAIL in inducing cell death in melanoma cells [15].

### 2.2. Caspase-3, Caspase-6, and Caspase-7

Caspase-3 and caspase-7 are executioner caspases that orchestrate the final stages of apoptosis. They facilitate cell death by degrading essential cellular proteins. Caspase-3, in particular, plays a pivotal role in this process by cleaving and activating other caspases, including caspase-6, caspase-7, and caspase-9 [22].

Liu et al. investigated the expression of active caspase-3 in melanoma cells and its implications for tumor behavior [23]. Their study revealed that active caspase-3, when triggered by proapoptotic stress factors, can induce migration in various cancer cell types. Notably, caspase-3 activation does not always lead to cell death; instead, it may enhance tumor aggressiveness. The researchers observed significant expression of activated caspase-3 in non-apoptotic melanoma cells, correlating with increased metastatic potential. Tumors exhibiting higher levels of activated caspase-3 were associated with a higher metastatic rate [23]. Additionally, these melanomas showed a greater prevalence of vascular mimicry (VM) networks. Furthermore, Liu et al. found that treatment with caspase-3 inhibitors significantly reduces levels of active matrix metalloproteinase 2 (MMP-2). They proposed that basal levels of active caspase-3 in cancer cells cleave pro-MMP-2 to its active form, promoting cell migration, tumor aggression, metastasis, and VM formation. Importantly, the expression of active caspase-3 was found to be independent of the patient’s sex, age, or tumor size [23].

Donato et al., in their publication, analyzed melanoma recurrence after cytotoxic therapy in terms of caspase-3 involvement [24]. There is a hypothesis according to which cytotoxic treatment activates paracrine signaling events that promote the growth of surviving melanoma cells [24]. They showed that dying melanoma cells activate the growth of viable tumor cells. This occurs via caspase-3. Conventional therapies often lead to the enhancement of apoptosis during the treatment process. In contrast, Donato et al. showed a counterintuitive conception of cancer cell biology. They confirmed that radiation and vemurafenib activate caspase-3 in melanoma cells and that dying melanoma cells treated with radiotherapy or vemurafenib stimulate the growth of viable tumor cells in vitro and in vivo. They showed that reducing caspase-3 activity using short hairpin RNA (shRNA) caspase-3 (dominant negative caspase-3 gene) or genetic deletion of caspase-3 in mouse embryonic fibroblasts (MEFs) leads to a reduction in the number of viable tumor cells.

Prostaglandin E2 (PGE2) plays a role in the caspase-3-mediated stimulation of the growth of viable melanoma cells by dying cells. The authors suggest that the modern therapy for superficial forms of melanoma is a combination of radiation and a topical caspase-3 inhibitor. For metastatic tumors, a combination of caspase-3 inhibitors with radiotherapy and/or vemurafenib treatment may also be beneficial [24].

Interleukin-1 Receptor-Associated Kinase (IRAK) is a family of serine–threonine kinases that play a crucial role in innate immune signaling pathways, particularly in response to interleukin-1 (IL-1) and Toll-like receptors (TLRs), which are essential for pathogen detection and initiating immune responses. The IRAK family includes several members, such as IRAK-1, IRAK-2, IRAK-M (also known as IRAK-3), and IRAK-4, each with distinct functions in immune signaling. IRAK-M specifically acts as a negative regulator of TLR signaling, preventing excessive immune activation and thereby limiting harmful inflammation [25]. Targeting IRAK signaling pathways is being explored as a therapeutic approach in cancer treatment. Geng et al. investigated the role of IRAK-M in the elimination of melanoma cells, demonstrating that the genetic and epigenetic modulation of IRAK-M can initiate apoptosis. This process involves the recruitment and degradation of calpastatin, an endogenous inhibitor of calpain. Subsequently, calpain activity is stimulated, triggering caspase-3-dependent, but caspase-8- and caspase-9-independent, apoptosis. Compounds that induce IRAK-M expression have been identified, with several FDA-approved drugs among them. These studies are paving the way for the development of effective targeted cancer therapies, where the therapeutic efficacy of certain approved drugs is closely linked to their ability to induce IRAK-M expression [25]. Consequently, IRAK-M is being investigated as a potential therapeutic target. Inhibiting IRAK-M activity could enhance melanoma cell susceptibility to apoptosis and improve the effectiveness of cancer therapies [25].

One study showed increased expression of activated caspase-6 in both primary and metastatic melanoma tumors. This is consistent with decreased levels of AP-2α and receptor tyrosine kinase c-kit, transcription factors whose loss is associated with melanoma progression [26]. Metastatic tumors additionally showed increased expression of activated caspase-3 [26]. AP-2α is cleaved by activated caspases (especially caspase-6) and subsequently degraded by the proteasome during TNF-α-induced apoptosis. In contrast, the presence of caspase-6 combined with the loss of AP-2α in primary melanomas and metastases may reflect excessive degradation of this protein by activated caspases. Epigenetic downregulation of AP-2α by activated caspase-6 may lead to impaired transcriptional control of various important proteins regulating cell adhesion, invasion, proliferation, and apoptosis [26].

Epigallocatechin gallate (EGCG), the main polyphenolic compound found in green tea, exhibits potential anticancer properties, including effects against melanoma. Research on the role of EGCG in melanoma has uncovered several key mechanisms through which it exerts its antiproliferative and proapoptotic effects [27]. Nihal et al. investigated the antiproliferative effects of EGCG using human melanoma cell lines. Melanocytes and melanoma cells typically exhibit high levels of the anti-apoptotic caspase inhibitor Bcl-2, with impaired apoptosis being a crucial factor in cancer development. Upon EGCG administration, the researchers observed a decrease in the levels of the anti-apoptotic protein Bcl-2; an increase in the pro-apoptotic protein Bax; and the activation of caspase-3, caspase-7, and caspase-9. Specifically, in amelanotic malignant melanoma cells, EGCG led to a significant increase in active caspase-3 and caspase-9, while caspase-7 levels were only slightly elevated. Conversely, in metastatic melanoma cells, EGCG significantly increased caspase-7 expression, with only a slight elevation in caspase-3 and caspase-9 levels. These findings suggest that in melanoma cells, EGCG induces apoptosis through the activation of caspases, which is modulated by alterations in Bcl-2 family proteins and associated apoptotic events [27].

Sun et al. explored the use of biomimetic nanoparticles to induce pyroptosis as a strategy for combining chemotherapy and immunotherapy against melanoma cells. In their study, they synthesized a biomimetic nanocarrier, zeolitic imidazolate framework-8 (ZIF-8), to deliver the chemotherapeutic agent oxaliplatin (OXA) and the immune modulator imiquimod (R837). Oxaliplatin activated caspase-3, initiating the degradation of intracellular proteins and thereby creating an inflammatory tumor microenvironment. The release of R837 from the nanoparticles further enhanced this inflammatory immune environment, leading to systemic anti-tumor immunity [28]. A recent study by Lee et al. identified the overexpression of Dock180 and Elmo1 in melanoma cells compared to healthy skin tissue. Dock180, a member of the Dedicator of Cytokinesis (DOCK) protein family, plays a critical role in the reorganization of the cellular cytoskeleton. Engulfment and Cell Motility Protein 1 (Elmo1) is an essential component of the signaling pathway associated with Dock180. The inhibition of Dock180 and Elmo1 led to reduced cell viability and induced apoptosis. This inhibition also downregulated Bcl-2, caspase-3, and PARP, while upregulating Bax, PUMA, cleaved caspase-3, and cleaved PARP [29]. BRAF inhibitors, such as vemurafenib and dabrafenib, are effective treatments for melanoma with BRAF mutations. However, these inhibitors are ineffective in BRAF wild-type melanoma, which lacks BRAF mutations [29]. For BRAF wild-type melanoma, new targeted therapies are being explored, particularly those involving the mitogen-activated protein kinase (MAPK) pathway. Inhibitors targeting MAPK pathways downstream of ERK1/2, as well as inhibitors of anti-apoptotic Bcl-2 proteins, like Mcl-1, are being investigated as alternative treatments [30,31]. Vemurafenib and the ERK inhibitor SCH772984, when used alone, demonstrated only limited efficacy in melanoma cell lines. However, the combination of SCH772984 and S63845 resulted in significant activation of caspases, including initiator caspase-8 and/or caspase-10. In the caspase cascade, these initiator caspases activate downstream effector caspases, such as caspase-3 and caspase-7. Activated effector caspases then cleave various substrates, including poly(ADP-ribose) polymerase (PARP), leading to the execution of apoptosis. This process is marked by histone H2AX phosphorylation, loss of the mitochondrial membrane potential, and the release of cytochrome c. The critical role of caspases in this process was confirmed by the pan-caspase inhibitor, which effectively blocked apoptosis induction and cell viability loss. In summary, the combined inhibition of ERK and Mcl-1 shows promising efficacy in both BRAF-mutant and wild-type melanoma cells, offering a potential new strategy to overcome drug resistance [31]. In response to cellular stress, such as DNA damage or oncogene activation, p53 is stabilized and activated. The p53 protein plays a central role in regulating genes essential for the cellular stress response, particularly in apoptosis. The p53 protein increases the expression of pro-apoptotic proteins, like Bax, PUMA, and Noxa, which promote the release of cytochrome c from mitochondria—a pivotal step in the intrinsic (mitochondrial) apoptotic pathway. Once in the cytoplasm, cytochrome c binds with Apaf-1, ATP, and procaspase-9 to form the apoptosome, where procaspase-9 undergoes autocatalytic activation. Activated caspase-9 then triggers a caspase cascade, notably activating caspase-3, which executes apoptosis by cleaving various cellular substrates [32]. Both p21 and p27 are recognized as tumor suppressor proteins with dual roles in cell protection. They can delay or inhibit apoptosis, allowing cells time to repair damage. However, if repair mechanisms fail, p21 and p27 can activate apoptosis by interacting with pro-apoptotic proteins, leading to the activation of caspases. The loss of function in these proteins can result in uncontrolled cell growth [33]. Pharmacological regulation of p27 can induce p27-dependent cell cycle arrest, enhancing the therapeutic efficacy of anticancer drugs [34]. Xie et al. demonstrated the inhibitory effect of artichoke extract on melanoma progression, providing evidence of increased caspase-3 activity, elevated p21 and p27 levels, and decreased CDK4 expression [35]. Furthermore, the expression of p27 has been directly correlated with melanoma progression [36]. Frohlich et al. studied patients with metastatic melanoma, showing that p53 activation increases sensitivity to BRAF inhibitors in cells with high p21 expression. This finding suggests a potential therapeutic strategy to improve treatment outcomes in patients with elevated p21 levels by combining BRAF inhibitors with drugs that enhance p53 activity [37]. Additionally, Kichina et al. described the role of p21-activated kinase 1 (PAK1) in reducing cell sensitivity to apoptosis. Inhibiting PAK1 could enhance the effectiveness of melanoma therapies, particularly in overcoming resistance to apoptosis [38].

### 2.3. Caspases-2, Caspase-8, Caspase-9, Caspase-10, and Caspase-12

Caspase-2, caspase-8, caspase-9, and caspase-10 are critical initiators of apoptosis [39,40]. Among these, caspase-2 is known to initiate apoptosis and may trigger a caspase cascade, leading to cell death in melanoma. Caspase-2 plays a pivotal role in apoptosis initiation, particularly in melanoma. Interferon beta (IFN-beta) is one of the most effective agents inducing melanoma cell apoptosis. IFN-beta-mediated apoptosis is largely dependent on the activation of tumor-necrosis-factor-related apoptosis-inducing ligand (TRAIL) [41]. Kamiya et al. demonstrated that IFN-beta induces apoptosis through increased activity of caspase-2 and caspase-3, with reduced efficacy when these caspases are inhibited. This suggests that caspase-2 regulation is crucial for the effectiveness of IFN-beta therapy and could potentially enhance melanoma cell sensitivity to chemotherapy [41].

In melanoma cells resistant to other therapies, such as docetaxel, caspase-2 can activate the mitochondrial apoptotic pathway, including the pro-apoptotic protein Bax. Mhaidat et al. found that docetaxel induces apoptosis in TRAIL-resistant melanoma cells through caspase-2 activation, which leads to mitochondrial changes and the subsequent activation of caspase-9 and caspase-3, alongside PARP degradation. These findings suggest a mitochondrial pathway for caspase-2-mediated apoptosis [42]. Jangi et al. similarly observed that caspase-2-dependent apoptosis in melanoma involves the mitochondrial pathway [43].

Caspase-8 is essential for apoptosis via death receptor pathways. Its activation in melanoma may influence the response to apoptosis-inducing therapies [44]. Inhibitor of Growth Family Member 4 (ING4) plays a role in regulating cellular growth, differentiation, and stress responses. Reduced ING4 expression in melanoma compared to normal nevi is associated with enhanced melanoma cell proliferation. The overexpression of ING4 can inhibit melanoma growth and induce apoptosis through the mitochondrial pathway by increasing Fas expression, which activates caspase-8. Active caspase-8 then triggers apoptosis via caspase-3 [44]. In conclusion, ING4 may play an anti-tumor role in melanomas through the Fas/caspase-8 apoptosis pathway, including inhibiting melanoma cell proliferation and growth and inducing apoptosis [44].

The findings also suggest an important role of caspase-8 and caspase-10 in the response to cytotoxic treatment, and caspase-8 may be a potential marker of the response to treatment [45]. Studies have shown that anti-apoptotic proteins of the Bcl-2 family are overexpressed in MM and may prompt resistance to apoptosis [45]. Caspase-10, like caspase-8, is linked to death receptor pathways. It relies on caspase-9 for full activation of the apoptotic pathway. Studies indicate that caspase-8 and caspase-10 are important in responding to cytotoxic treatments, with caspase-8 potentially serving as a marker for treatment efficacy. Keuling et al. demonstrated that melanoma cells with high levels of active caspase-8 and Bid show increased sensitivity to ABT-737, a treatment that induces significant cleavage of caspase-8 and Bid. Conversely, no correlation was observed between caspase-10 levels and the treatment response [45]. However, studies have also demonstrated a mechanism for the death-receptor-independent activation of caspase-8/-10 in response to several anticancer agents, including camptothecin, paclitaxel (Taxol), and etoposide [46,47,48]. In such cases, active caspase is thought to act as part of a feedback loop to enhance the caspase cascade and amplify apoptotic signals. The loss or inhibition of caspase-8 or caspase-10 significantly reduces the effectiveness of these therapies. Thus, the death-receptor-independent activation of caspase-8/caspase-10 appears to be an important apoptotic mechanism for some chemotherapeutic agents. The inhibition of caspase-8 or caspase-10 (as well as disabling caspase-8) significantly reduces the efficacy of treatment, suggesting that this downstream pathway may play an important role in the response of melanoma cells to combination therapy [45].

Caspases also play roles in inhibiting autophagy and redirecting cells toward apoptosis. You et al. explored the use of an arginine-degrading enzyme in melanoma cells lacking ASS expression. They found that caspase-8 inhibitors could completely prevent the cleavage of Beclin-1 and Atg5, essential for autophagy. Caspase-3, caspase-6, caspase-9, and caspase-10 inhibitors had partial effects [49]. Functional polymorphisms in CASP8 and CASP10 may also influence melanoma susceptibility. Li et al. identified associations between CASP8 D302H, CASP8 -652 6N, and CASP10 I522L polymorphisms with melanoma risk, suggesting these variants as potential biomarkers [50].

In addition, Wu et al. showed that the activation of caspase-8, caspase-9, and caspase-3 contributes to the apoptosis of human A375-S2 melanoma cells in response to the administration of the natural product shikonin [51]. Decreased levels of Bcl-2 expression and elevated levels of Bax expression are associated with elevated levels of p53 and caspase-8 and caspase-9 expression, leading to the activation of downstream caspase-3 in this mechanism [51].

Caspase-12 is involved in apoptosis related to endoplasmic reticulum stress and inflammatory responses. Kalai et al. found that IFN-gamma stimulates caspase-12, but in IFN-γ-treated melanoma cells, increased expression of inflammatory caspases (caspase-1, caspase-11, and caspase-12) and decreased expression of apoptotic caspases (caspase-3 and caspase-9) suggest an alternative apoptotic cascade. Despite its role in apoptosis, caspase-12′s involvement in ER-stress-mediated cell death in melanoma remains unclear, as melanoma cells can die independently of caspase-12 presence [52].

### 2.4. Caspase-14

Caspase-14, a member of the conserved family of aspartate-specific proteinases, is primarily expressed in the suprabasal layers of the epidermis and hair follicles. Denecker et al. described the role of caspase-14 in epidermal maturation through the proteolytic processing of filaggrin [53,54]. In addition, the proteolytic activation of caspase-14 has been implicated in the formation of the stratum corneum, leading to the involvement of caspase-14 in keratinocyte differentiation and keratinization. In the epidermis, caspase-14 is responsible for the processing of profilaggrin. Mice deficient in caspase-14 exhibit increased sensitivity to cyclobutane-pyrimidine dimer formation following UVB irradiation, leading to heightened UVB-induced apoptosis. Thus, caspase-14 plays a critical role in the ability of the stratum corneum to protect itself against UVB radiation [54]. Wang et al. demonstrated that caspase-14 is expressed in melanoma cells and melanocytes. They found that treatment with chemotherapeutic agents, such as camptothecin and cisplatin, or radiotherapy is more effective in cells with lower levels of caspase-14 expression. In malignant melanoma cells, caspase-14 is activated, and its expression level affects the sensitivity of melanoma to chemotherapeutic agents [55].

## 3. Conclusions

In previous studies on the role of caspases in melanoma pathogenesis, it has been shown that the activation of caspases can induce cell death in this cancer, thereby inhibiting melanoma growth and progression. The deficiency or downregulation of caspases can impair the process of apoptosis, allowing melanoma cells to avoid cell death and continue to proliferate. Studies have shown that caspase-3 in particular can stimulate melanoma metastasis through the cleavage (cleavage) of specific cellular substrates. The cleavage of these molecules can enhance melanoma invasiveness and facilitate the spread of tumor cells into further regions. A summary of the contribution of proinflammatory caspases and apoptotic caspases to the pathogenesis of melanoma is presented in Table 1 and Table 2, respectively. The role of caspase in the pathogenesis of melanoma still needs further investigation.

## Figures and Tables

**Table 1:** Overview of the contribution of proinflammatory caspases to the pathogenesis of melanoma.

Type of Caspase	Potential Effect	References
Caspase-1	- It is involved in the development of melanoma metastasis. - There is a positive correlation between caspase-1 levels and tumor mass, as well as resistance to treatment.	[14,17]
Caspase-1, caspase-5	- Cleaved caspase-1 activates constitutively the NALP3 inflammasome in human melanoma cells. - Activated fragments of caspase-1 (p20) and caspase-5 (p20) are observed in lysates of human metastatic melanoma cells without IL-1α stimulation. - Activated caspases mediate IL-1β secretion in human metastatic melanoma cells (autoinflammation mediated by IL-1 contributes to the development and progression of human melanoma).	[19]
Caspase-12	- The inducible expression of caspase-12 leads to proteolytic processing of the enzyme and the apoptosis of melanoma cells.	[52]
Caspase-14	- Cells with lower levels of caspase-14 expression are more sensitive to treatment with chemotherapeutic agents or radiotherapy than cells with higher levels of caspase-14 expression.	[55]

**Table 2:** Summary of the role of apoptotic caspases in the pathogenesis of melanoma.

Type of Caspase	Potential Effect	References
Caspase-2	- It plays a significant role in initiation of the processes of programmed cell death in MM cells. - It can be activated by IFN-beta (could be a potential predictor of IFN-beta treatment efficacy). - It initiates mitochondrial-dependent apoptosis through direct or indirect activation of Bax protein. - It may play a key role in the apoptotic response of melanoma cells to certain chemotherapeutic agents.	[39,40,41,42,43]
Caspase-3	- Activated caspase-3 is expressed in non-apoptotic melanoma cells and contributes to melanoma migration, invasion, metastasis, and VM formation. - It mediates the mechanism of the growth of living tumor cells after cytotoxic treatment in dying melanoma cells. - The induction of IRAK-M expression using genetic or epigenetic modifications initiates apoptosis.	[23,24,25]
Caspase-3, caspase-4	- They contribute to TRAIL-induced apoptosis and are associated with the induction of ER stress by TRAIL in melanoma cells. - Caspase-3 inhibition blocks caspase-4 activation, while caspase-4 inhibition attenuates TRAIL-induced caspase-3 activation.	[19]
Caspase-3, caspase-6	- The increased expression of activated caspase-6 in the skin compartment of primary and metastatic tumors corresponds to decreased levels of AP-2α and c-kit, while the expression of activated caspase-3 characterizes metastatic cells.	[26]
Caspase-3, caspase-7, caspase-9	- EGCG treatment of melanoma cells results in increased levels of pro-apoptotic Bax and the activation of caspase-3, caspase-7, and caspase-9.	[27]
Caspase-8, caspase-9, caspase-10	- Activated caspase-8, caspase-9, and caspase-10 are involved in apoptotic cell death and the cytotoxic effect of the combined treatment of melanoma. - The genotypes of CASP8 D302H and CASP8 -652 6N variants are associated with melanoma risk in single-locus analysis, and the combined genotypes of CASP8 and CASP10 polymorphisms may be biomarkers of melanoma susceptibility. - The SNP variants CASP8 D302H, CASP8 -652 6N, and CASP10 I522L and their haplotypes influence melanoma risk in multivariate analysis.	[45]
Caspase-8	- ING4 may play an anti-tumor role in melanomas through the Fas/caspase-8 apoptosis pathway.	[44]
Caspase-3, caspase-8, caspase-9	- The activation of caspase-3, caspase-8, and caspase-9 is involved in inducing apoptosis by shikonin	[51]

## Data Availability

Not applicable.
